# Supplementary material for: Mitomycin C eliminates cyanobacterial transcription without detectable prophage induction in a Microcystis-dominated harmful algal bloom in Lake Erie
Source: Microbiol Spectr. 2025 Apr 9;13(5):e02872-24. doi: 10.1128/spectrum.02872-24 (PMC12054034; doi:10.1128/spectrum.02872-24)
Supplement: Supplemental Figures — Figures S1 to S6. [file spectrum.02872-24-s0001.pdf]

Supplemental Information for:

Mitomycin C eliminates cyanobacterial transcription without detectable prophage induction in a *Microcystis*-dominated harmful algal bloom in Lake Erie

Robbie M. Martin[a], Elizabeth R. Denison[a], Helena L. Pound[a], Ellen A. Barnes[a], Justin D. Chaffin[b], Steven W. Wilhelm[a#]

[a]Department of Microbiology, University of Tennessee at Knoxville, Knoxville, TN, USA
[b]F.T. Stone Laboratory, Ohio Sea Grant, and The Ohio State University, Put-In-Bay, OH, USA

Running Head: Mitomycin C eliminates transcription in Cyanobacteria

#Address correspondence to Steven W. Wilhelm, wilhelm@utk.edu

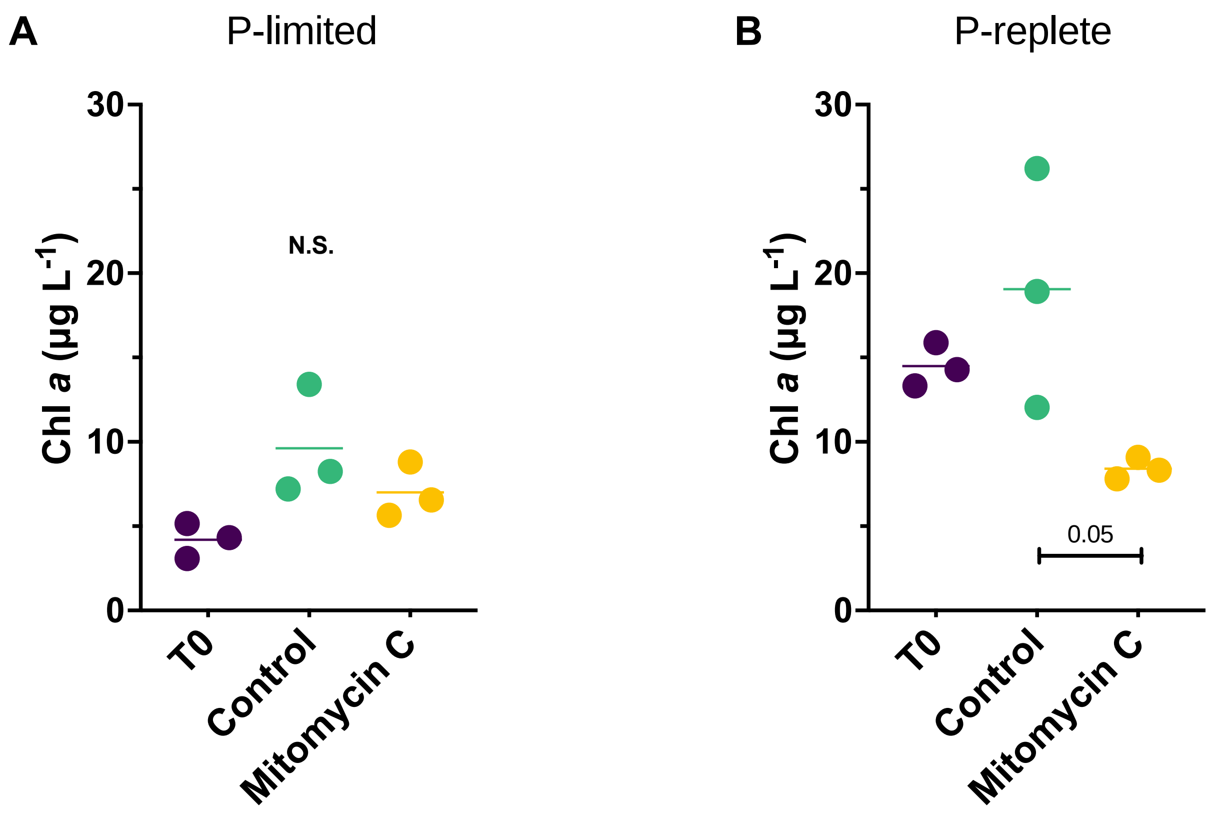

Fig. S1. Chlorophyll *a* response by treatment. A) P-limited experiment. B) P-replete experiment.

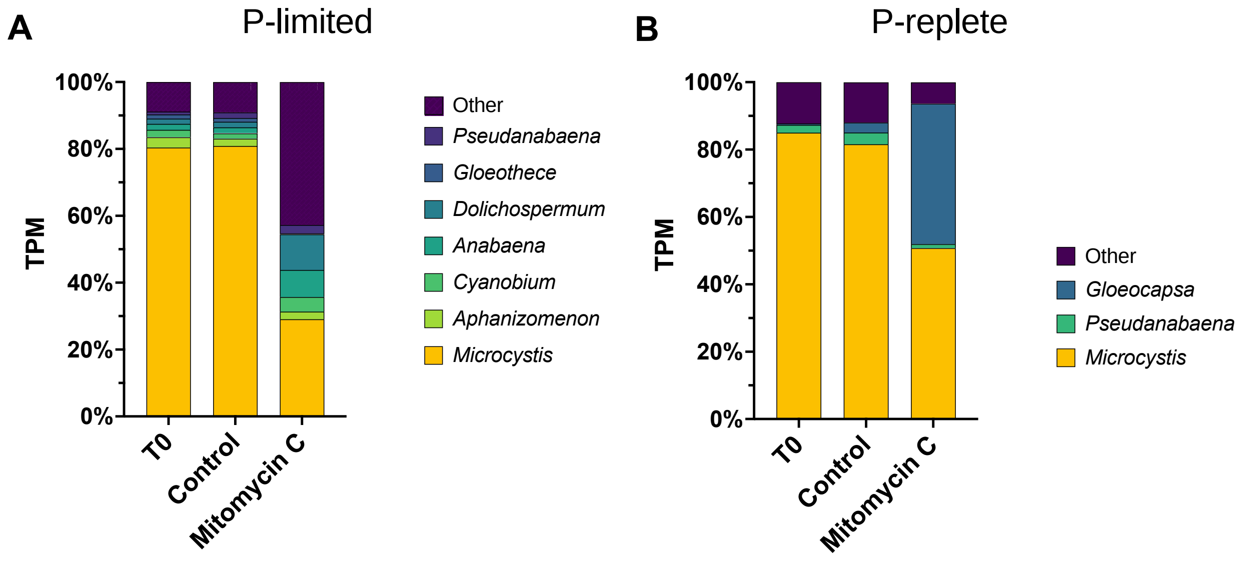

Fig. S2. Transcription activity of major genera of Cyanobacteria Phylum by treatment as a percent of total cyanobacterial transcription. A) P-limited experiment. B) P-replete experiment.

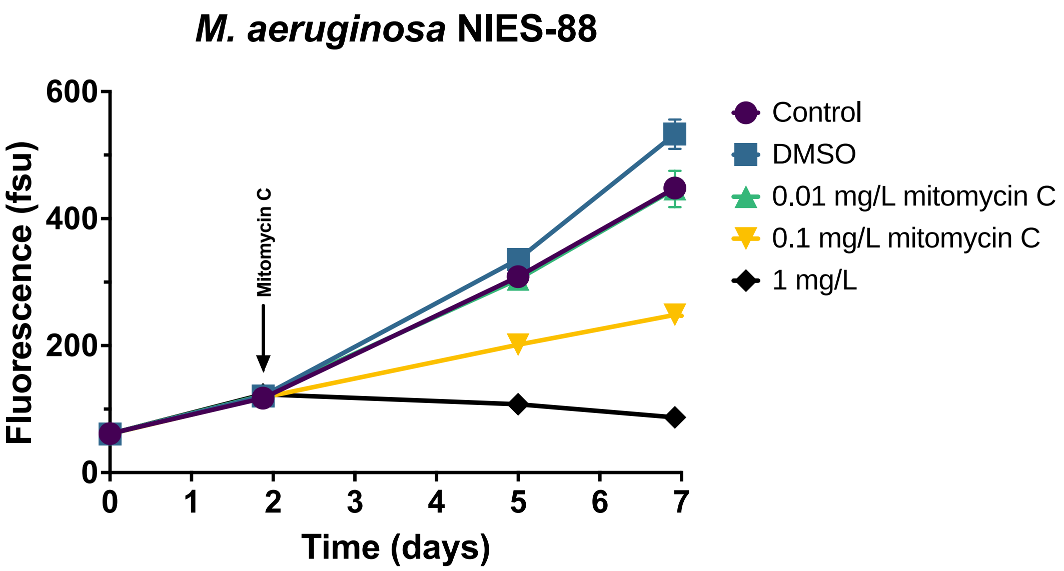

Fig. S3A. Dose-dependent growth response of *Microcystis aeruginosa* NIES-88 to mitomycin C.

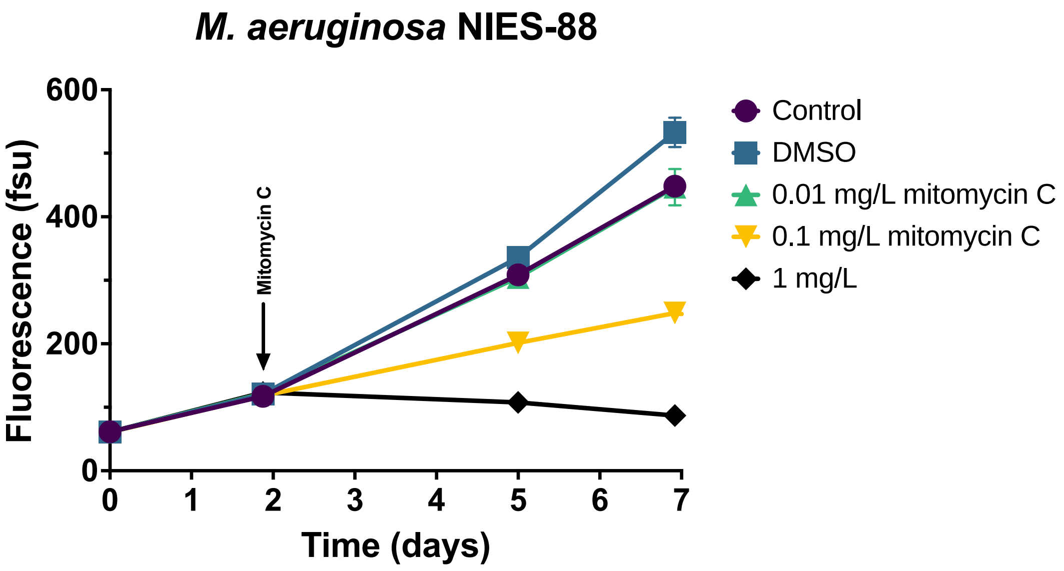

Fig. S3B. Dose-dependent growth response of *Microcystis aeruginosa* NIES-88 to mitomycin C.

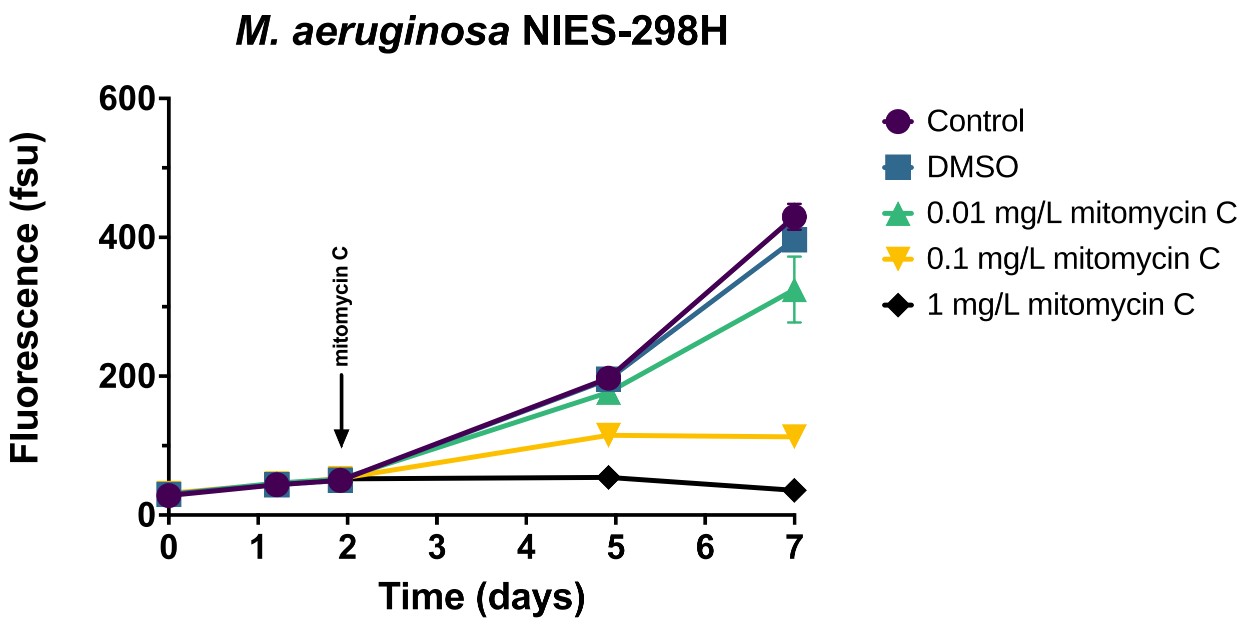

Fig. S4. Dose-dependent growth response of *Microcystis aeruginosa* NIES-298H to mitomycin C.

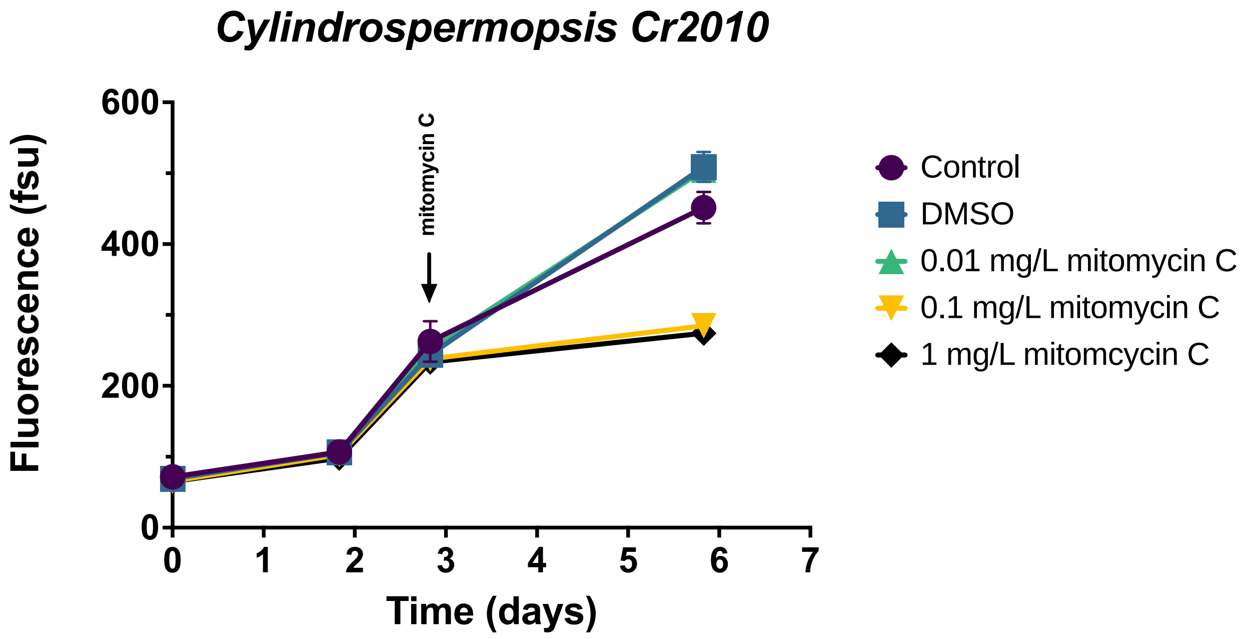

Fig. S5. Dose-dependent growth response of *Raphidiopsis (Cylindrospermopsis) raciborskii* Cr2010 to mitomycin C.

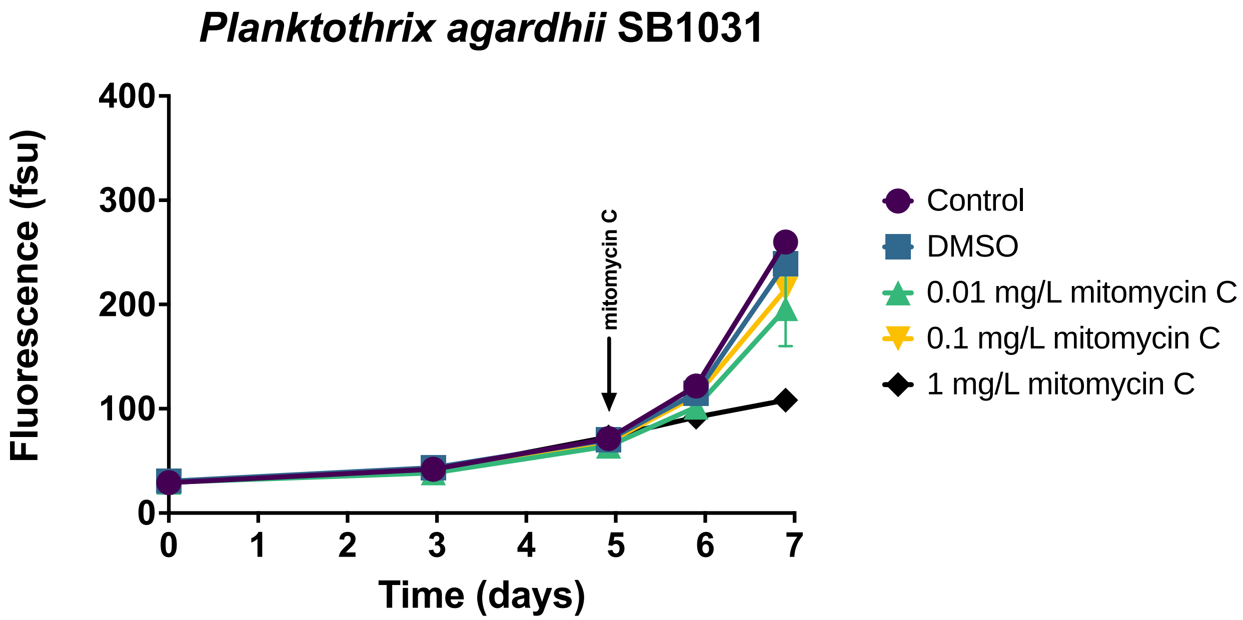

Fig. S6. Dose-dependent growth response of *Planktothrix agardhii* SB1031 to mitomycin C.

Table S1. Submitted as separate Excel spreadsheet.

Table S2. Submitted as separate Excel spreadsheet.
